# Maxillofacial Microvascular Free-Flap Reconstructions in Pediatric and Young Adult Patients—Outcomes and Potential Factors Influencing Success Rate

**DOI:** 10.3390/jcm13072015

**Published:** 2024-03-30

**Authors:** Dominika Lech, Jeremi Matysek, Robert Maksymowicz, Cyprian Strączek, Robert Marguła, Łukasz Krakowczyk, Marcin Kozakiewicz, Krzysztof Dowgierd

**Affiliations:** 1 Department of Clinical Pediatrics, Head and Neck Surgery Clinic for Children and Young Adults, University of Warmia and Mazury, Żołnierska 18a Street, 10-561 Olsztyn, Poland; dominika.lech@student.uwm.edu.pl (D.L.); jeremi.matysek@student.uwm.edu.pl (J.M.); robert.maksymowicz@student.uwm.edu.pl (R.M.); cyprian.straczek@student.uwm.edu.pl (C.S.); robert.margula@student.uwm.edu.pl (R.M.); 2 Oncological and Reconstructive Surgery Clinic, Branch of National Oncological Institute in Gliwice, Maria Sklodowska-Curie Institute—Oncology Centre (MSCI), Ul. Wybrzeze Armii Krajowej 15, 44-100 Gliwice, Poland; 3 Department of Maxillofacial Surgery, Medical Univeristy of Lodz, 113 Żeromskiego Str., 90-549 Lodz, Poland; marcin.kozakiewicz@umed.lodz.pl

**Keywords:** pediatric, free flap, microvascular reconstruction, head and neck, outcomes, success rate, complications

## Abstract

**Background**: Maxillofacial microvascular free-flap reconstructions are significant interventions in the management of congenital defects, traumatic injuries, malignancies, and iatrogenic complications in pediatric and young adult patients. Craniofacial disorders within this demographic can result in profound functional, cosmetic, and psychosocial impairments, highlighting the critical need for thorough investigation into factors that may influence procedural success and postoperative quality of life. This retrospective chart review aims to examine the outcomes and potential influencing factors, aiming to offer valuable insights into optimizing the effectiveness of these reconstructions and improving patient outcomes. **Methods**: A single head and neck surgical team performed all the included 136 procedures. Demographic and surgical patient data were recorded. Type of transfer performed in each recipient site and major complications were analyzed. Relevant influencing factors, such as age, gender, and etiology of defect were determined using the ANOVA test and χ^2^ test of independence. **Results**: The results indicate a 90% success rate. No significant relationship was found between the incidence of total flap loss and patient age, etiology, or graft source. The maxillary reconstructions showed a higher incidence of total flap loss compared to mandibular reconstructions (11 vs. 3 cases). **Conclusions**: Despite the high success rate, the findings underline the necessity for further research to validate these observations and enhance surgical methods for pediatric and young adult patients.

## 1. Introduction

Head and neck disorders in pediatric and young adult patients can result in significant functional and cosmetic deformities [1], originating from causes such as congenital defects, traumatic injuries, malignancies, and iatrogenic complications. Microvascular free-flap reconstructions have become essential in addressing these complex deformations, transforming the field of head and neck reconstruction by enabling the transfer of reliable bone and soft tissue from distant sites using microsurgical techniques [2]. In the context of pediatric and young adult patients, however, there exists a significant gap in detailed research explaining the specific impacts and nuances of these procedures [1,3,4,5]. While previous studies have explored potential determinants influencing the success rates of microvascular free-flap reconstructions [1,3,6,7], a consensus regarding these factors remains unclear, indicating the need for further investigation.

The physiological and developmental characteristics unique to youth require specialized approaches different from those used in adult populations [8]. By examining variables such as age, sex, etiology of the maxillofacial defect, graft source, and recipient site location, this research seeks to understand the relationship between these factors and surgical outcomes. Through a detailed analysis of a cohort comprising 136 pediatric and young adult patients who underwent maxillofacial microvascular free-flap reconstructions, this study aims to identify key determinants impacting surgical success. The findings are expected to provide a basis for future research aimed at improving the effectiveness and enhancing the post-surgical quality of life for pediatric and young adult patients undergoing these procedures [9].

## 2. Materials and Methods

This is a retrospective chart review from August 2011 to June 2023. Data were collected from the Maxillofacial Surgery for Children and Young Adults Division in the Head and Neck Clinic, Regional Specialized Children’s Hospital in Olsztyn, Poland. This study included patients from 1 to 25 years of age. A total of 136 procedures performed on 136 patients with complete medical records were analyzed. Patients were categorized by recipient site anatomical location, and major complications were recorded.

### 2.1. Procedures and Techniques

The free-flap auto-transplantation procedure began with the resection of pathology, resulting in tissue loss in the recipient site. Next, the flap was harvested from the donor site but remained connected to surrounding tissue by at least one artery and one vein. Simultaneously, the recipient site was surgically dissected to prepare the recipient artery, the facial artery, and vein, predominantly the facial vein, for anastomosis with the vascular pedicle of the free flap. The free flap was brought to the defect area and the vessels of the flap were anastomosed with the vessels of the recipient site, under the control of a microscope. After reconnection, the free flap was sutured to the defect, while the medical team monitored blood flow in the anastomosed vessels to ensure patency. Meanwhile, the donor site was primarily closed.

### 2.2. Terms

Iatrogenic etiology refers to cases where surgical interventions, initially intended to address a medical condition or trauma, inadvertently result in further complications or damage requiring microvascular free-flap reconstructive surgical intervention.

Lower limb nerve flap refers to a vascularized free flap containing skin, subcutaneous tissue with or without muscles and sural or tibial nerves acquired from the lower limb.

### 2.3. Data Collection and Statistical Analysis

Data for this study were extracted from electronic health records. A database was established for analysis. Recorded parameters included gender, age, etiology of the condition, recipient and donor sites, as well as postoperative complications.

The statistical analysis was performed using STATGRAPHICS Centurion 19 (StatPoint, Tulsa, OK, USA). The ANOVA test was utilized to determine relationships between age as a continuous variable and recipient site complications, etiology, and total flap loss. The χ^2^ test of independence was applied to assess relationships among categorical variables, including age groups, gender, recipient site complications, donor site, etiology, and the incidence of total flap loss. Age groups were categorized as follows: less than 5 years, 5 to 10 years, 11 to 15 years, 16 to 20 years, and over 20 years. A threshold of *p* < 0.05 was set to determine statistical significance.

## 3. Results

This study included 136 young patients who underwent microvascular free-flap reconstructions, comprising 76 females and 60 males. The median age was 14 years, ranging from 1 to 25 years. Table 1 accurately delineates the demographic and clinical data, illustrating gender distribution, etiology, recipient and donor sites, and recipient site complications. The predominant etiology of the underlying pathology was neoplastic in nature, accounting for 82 out of 136 cases (60.3%), followed by congenital defects in 39 cases (28.7%). The most frequently reconstructed sites were the maxilla (56 out of 136 cases, 41.2%) and mandible (55 out of 136 cases, 40.4%). The fibula (47 out of 136 cases, 34.6%) and iliac crest (44 out of 136 cases, 32.4%) were the most harvested flaps. Out of the 136 procedures performed, 122 resulted in successful free-flap survival, while 14 cases experienced total flap loss, yielding an overall success rate of 89.7%. Postoperative complications included total flap necrosis in 14 cases (10.3%), partial flap necrosis in 11 cases (8.1%), abscess formation in 4 cases (2.9%), and nerve palsy in 1 case (0.7%). The distribution of total flap necrosis was 11 in maxillary reconstructions and 3 in mandibular reconstructions. Within the maxillary reconstruction group, the total flap loss was distributed among donor sites as follows: five cases from the iliac crest (representing 20.8% of all iliac crest flaps transplanted to the maxilla), five from the fibula (35.7% of all fibular flaps to the maxilla), and one from the medial condyle of the femur (constituting 7% of all such flaps to the maxilla). Within the mandibular reconstruction group, the total flap loss was distributed among donor sites as follows: two cases from the fibula (6.1% of all fibular flaps to the mandible) and one case from the iliac crest (representing 5% of all such flaps to the mandible).

### 3.1. Patient’s Age at the Time of Surgery and Total Flap Loss

**Figure 1:**
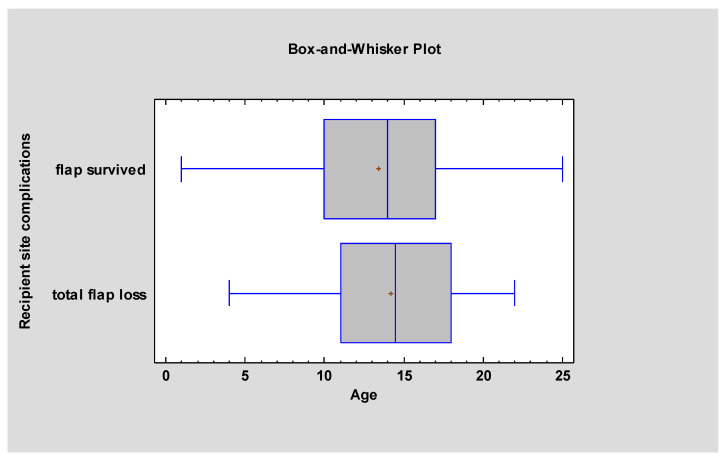
There is no age-dependent relationship of total flap loss.

Table 2 shows that the mean age at the time of microsurgical reconstruction was 13.5 (±4.98), with a median of 14 years. For patients who had a successful procedure, the mean age was 13.4 (±5.0), with a median of 14 years. In contrast, the mean age for those with flap failure was 14.2 (±4.95), with a median of 14.5 years. Statistical analysis indicated no significant age difference between the patients with flap survival and those with flap loss [F (4,131) = 0.33, *p* = 0.57], which is presented in Figure 1.

### 3.2. Gender, Etiology of the Underlying Pathology, and the Occurrence of Total Flap Loss

**Figure 2:**
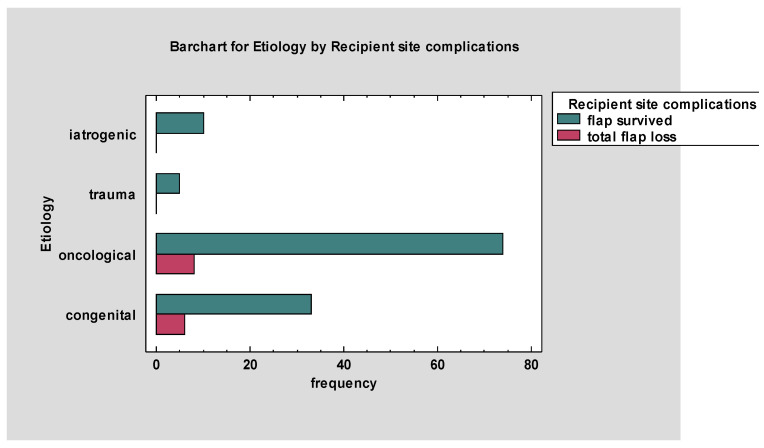
Numbers of total flap losses and flap survival by etiology of reconstruction.

Figure 2 provides an overview of the data. In evaluating the impact of etiology on the incidence of total flap loss and flap survival, the oncological group demonstrated a total flap loss in eight cases, which constituted 5.88% of all one hundred and thirty-six cases. The congenital etiologies had a lower incidence of total flap loss, with six cases representing 4.41% of all reconstructions performed. Both trauma and iatrogenic categories maintained a 100% flap survival rate with no instances of total flap loss. Statistical analysis revealed no significant differences in the incidence of total flap loss across etiology groups [χ^2^ (3, *N* = 136) = 2.84, *p* = 0.42].

Table 3 indicates that female patients underwent more flap transfers than male patients (*n* = 76 vs. *n* = 60) and experienced a higher incidence of total flap loss (*n* = 11 vs. *n* = 3). As a result, the success rate was lower among females (86%) than males (95%). However, statistical analysis did not reveal any significant differences between the genders in terms of flap survivability [χ^2^ (1, *N* = 136) = 3.26, *p* = 0.07].

### 3.3. Age and the Occurrence of Recipient Site Complications

**Figure 3:**
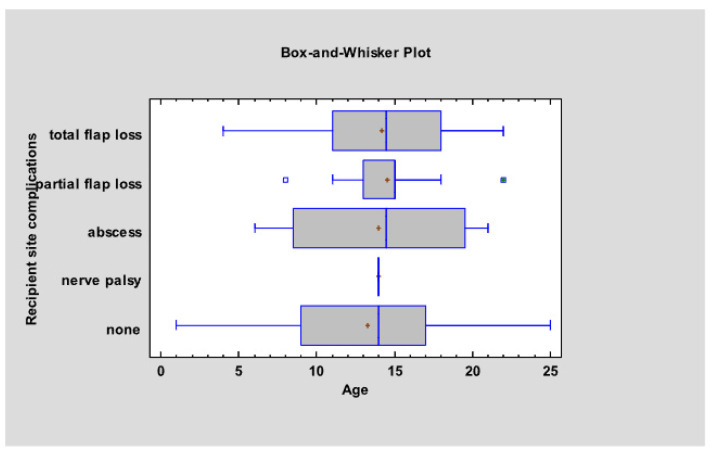
There is no age-dependent relationship of recipient site complications.

Figure 3 provides an overview of the data. No recipient site complications were recorded in 106 procedures. The mean age of patients without recipient site complications was 13.26 (±5.11) with a median age of 14 years. The most common complication in recipient site was total flap loss (*n* = 14) with a mean age at the time of procedure of 14.21 (±4.95) and a median age of 14.5 years. Eleven procedures resulted in partial flap loss with a mean age of 14.55 (±3.56) and patients’ median age of 15 years. The recipient site complication was abscess in four procedures, with a mean and median age at the time of surgery of 14.00 (±6.78) and 14.5 years, respectively. One procedure resulted in nerve palsy in a 14 y.o. patient. No significant relationship was found between age and recipient site complications [F (4, 131) = 0.26, *p* = 0.90].

### 3.4. Occurrence of Recipient Site Complications between Age Groups and Age Group Specific Success Rate

**Figure 4:**
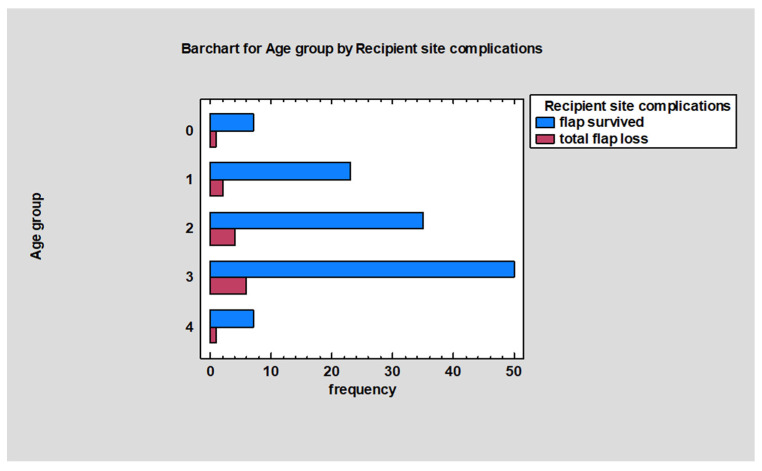
Number of procedures resulting in flap survival or total flap loss for age groups. Numbers on Y axis are labels of age groups (“0”= less than 5 y.o.; “1”= 6 to 10 y.o.; “2” = 11 to 15 y.o.; “3” = 16 to 20 y.o.; “4” = greater than 20 y.o.).

Table 4 provides a summary of recipient site complications categorized by age groups. The 16 to 20-year-old group had the highest incidence of recipient site complications, which also correlated with having the highest number of procedures and the highest number of cases resulting in total flap loss. The fewest complications were noted in patients under 5 years of age, with this group having only one case of total flap loss and no other documented complications. The group aged 6 to 10 years demonstrated the highest success rate at 92%. Statistical analysis revealed no significant differences in the incidence of recipient site complications across age groups [χ^2^ (16, *N* = 136) = 7.94, *p* = 0.95]. Additionally, Figure 4 indicated that there was no significant relationship between age groups and the number of total flap losses [χ^2^ (4, *N* = 136) = 0.24, *p* = 0.99].

### 3.5. Recipient Site, Recipient Site Complication and Flap Survival

Table 5 highlights a significant discrepancy in the incidence of total flap loss between the maxilla and mandible groups. The maxilla group, with the highest number of transplants (*n* = 56), also had the highest incidence of total flap loss (*n* = 11), resulting in a success rate of 80.4% for free-flap transplants in this group. In contrast, of the 55 free-flap transplantations to the mandible, only 3 resulted in total flap loss, yielding a success rate of 94.6% for mandibular reconstructions. The difference in total flap loss between the two sites was statistically significant (*p* < 0.05) [χ^2^ (4, *N* = 136) = 9.56, *p* = 0.049]. For the maxillary transplants, 24 (42.9%) flaps were harvested from the iliac crest, 14 (25%) from the fibula, 14 (25%) from the medial condyle of the femur, 3 (5.4%) from the anterolateral thigh, and 1 from the forearm. In the 55 mandibular transplants, there were 33 fibula flaps (60%), 20 (36.4%) flaps from the iliac crest and 2 (3.6%) flaps from the medical condyle of femur. In the groups undergoing soft tissue, orbital, and facial nerve microsurgical reconstruction, there were no instances of total flap loss, leading to success rates of 100% in these categories.

Table 6 indicates that the maxilla group experienced the highest number of recipient site complications, followed by the mandible and soft tissue groups. There were no reported complications for free-flap transfers to the orbital or facial nerve. Excluding total flap loss, the complication rate for free-flap transfers to the maxilla was 10.7%, while transfers to the mandible had a complication rate of 16.4%. The results of the χ^2^ test suggest no significant association between the recipient sites and the occurrence of complications [χ^2^ (4, *N* = 136) = 7.10, *p* = 0.13].

## 4. Discussion

This study conducted an extensive examination of maxillofacial microvascular free-flap reconstructions in a pediatric and young adult cohort, yielding significant insights into the success rates and factors influencing outcomes. The observed success rate of 89.71% in our study, while notable, is somewhat lower compared to the success rates typically reported in the existing literature, which often exceed 94% [1,3,4,5,9,10,11,12]. In a study by Liu et al. (2018) focusing on pediatric head and neck reconstruction, a higher success rate of 95.6% was reported [1]. However, it is essential to highlight the differences in the distribution of recipient sites between the two studies. Our research found the maxilla (55 out of 136 cases) and mandible (55 cases) to be the most common recipient sites, with the maxilla having the highest incidence of total flap loss. Notably, the success rate for mandibular reconstructions in our study was 94.55%, closely aligning with the higher success rates reported in the literature. In contrast, the study by Liu et al. primarily involved mandibular reconstructions (88 out of 135 cases), with only nine cases of maxillary reconstructions. Despite their conclusion of no significant relationship between recipient site and total flap loss, the predominance of mandibular reconstructions in their study, which aligns closely with the higher success rates in our mandibular cases, might partially explain the overall greater results observed in their findings.

In our analysis, we specifically examined the relationship between patient age and the incidence of total flap loss. It has been observed that children under ten years of age might be at a heightened risk of lower success rates in these procedures [1]. The potential underlying factors attributed to this finding include the reduced diameter of vasculature in younger patients, arterial vasospasms, and heightened complexity in performing surgical techniques on smaller anatomical structures. Regardless of these findings, our data did not demonstrate a significant relationship between patient age and the incidence of total flap loss. Interestingly, this result is consistent with another substantial study involving 102 patients, where a similar lack of relationship between age and surgical success in microvascular reconstructions was observed [13]. This parallel outcome in a separate large-scale study reinforces the notion that age, while an important consideration, may not be as critical a determinant of flap survival.

We investigated the potential relationship between patient gender and the incidence of total flap loss. Our examination revealed a borderline statistical significance (*p* = 0.071), suggesting a tentative yet not statistically validated trend towards a higher risk of total flap loss in female patients. However, given the marginal nature of this finding, it necessitates further investigation with an expanded pediatric sample size to establish a more definitive conclusion. The literature presents varied perspectives on the influence of gender in head and neck reconstructions. For example, Loupatatzi et al. identified female gender as one of the factors associated with increased complications in head and neck cancer reconstructions, alongside pre-operative radiation therapy and extended surgery duration [14]. In contrast, Rohleder et al. reported no significant gender-related differences in the postoperative outcomes of free-flap reconstructions in the head and neck region [15]. It is important to note, however, that these studies predominantly involved adult populations, with mean ages notably above the pediatric range, and thereby limiting the applicability of their findings to a younger demographic.

A striking finding was the higher incidence of total flap loss in maxillary reconstructions compared to mandibular ones. Specifically, the maxilla experienced 11 cases of total flap necrosis out of 55 reconstructions, translating to a success rate of 80.36%, markedly lower than the 94.55% rate observed for mandibular reconstructions. This contrast becomes even more pronounced when compared to adult maxillary reconstruction success rates, which typically hover around 95% in the literature [16,17]. However, it aligns more closely with recent findings in pediatric patients, such as those reported by Burns et al. (2023), who observed a 23% total flap loss in pediatric maxillary reconstructions [18].

The absence of any total flap loss instances in reconstructions involving soft tissues, orbital regions, and facial nerves is noteworthy. The results are consistent with the noted trend that flaps incorporating bone have a nearly five-fold higher failure rate compared to those consisting entirely of soft tissue. This is likely attributable to the fact that in bone defect reconstructions, the positioning of both the flap and its pedicle is dictated by the bony defect, offering limited flexibility for alteration [19].

Moreover, the findings of our study hold potential utility in empowering both patients and their parents to make more informed decisions regarding free-flap microvascular reconstruction. It is an ethical obligation for physicians to provide comprehensive information to patients, encompassing diagnosis, planned treatment, postoperative complications, and success rates. Agozzino et al.’s study has contributed valuable insights into patient satisfaction and the frequency of legal claims concerning surgical procedures. The research revealed that patients who received both written consent and oral information about procedures exhibited higher satisfaction with surgical treatment compared to those who received written consent alone. Remarkably, 19.6% of individuals receiving both written and oral information reported feeling influenced to varying degrees. Notably, information regarding postoperative complications and success rates received limited attention from physicians. However, when conveyed, such information correlated with increased satisfaction with treatment and reduced patient’s anxiety [20]. These findings underscore the importance of effective communication, providing reliable data on postoperative complications and success rates in the context of free-flap microvascular reconstructions. This could potentially enhance the satisfaction of patients and their parents while concurrently reducing the incidence of legal claims. Nevertheless, the study is subject to certain limitations. Primarily, it was conducted in general surgery units in Italy, specifically on adult patients capable of legally consenting to surgery. Consequently, the generalizability of these findings to pediatric settings is restricted to patients’ parents. Additionally, the study relied on face-to-face interviews conducted several days after patients had received written consent, introducing a potential risk of recall bias.

In our clinical practice, maxillofacial free-flap reconstructive surgeries are often necessitated by various etiologies, including oncological, traumatic, and congenital factors. These procedures not only address medical needs, such as tumor resections, but also significantly enhance craniofacial function, repair defects, and mitigate facial deformities. However, it is crucial to recognize that these surgeries invariably alter the patient’s facial appearance, underscoring the importance of properly informing both patients and parents about this fact. Parental involvement in decision-making regarding pediatric reconstructive surgery is pivotal, as some advocate for proactive surgical intervention, while others suggest waiting until the child can actively participate in the decision-making process [21]. Incorporating intervention strategies, such as psychological support before and after surgery, as well as potential corrective cosmetic procedures, enables the effective management of their psychological burdens postoperatively and may help to tone down the negative psychosocial consequences. In particular, for appearance-sensitive adolescents, counseling pre- and postoperatively could be required to prepare them for the resultant changes. This aligns with findings from studies on head and neck reconstructions, which highlight the significant impact on patients’ psychological well-being, especially among vulnerable groups such as women with a history of anxiety or depression [22,23]. Similarly, research on patients with tongue cancer undergoing resection procedures emphasizes the variations in quality of life and psychological status, with more extensive surgeries often resulting in worse outcomes [24]. Therefore, it is critical for healthcare professionals to advocate for patients considering surgery, facilitate informed decision-making, and mitigate emotional and social obstacles by openly discussing potential challenges pre-operatively, developing coping mechanisms, and educating parents and peers to reduce post-surgery psychological distress [21].

Despite advancements in reconstructive surgery, the management of complications following flap failure remains an area with significant gaps in understanding and exploration [25]. In our practice, the approach entails the removal of the necrotic tissue flap followed by reoperation. Additionally, thorough discussions with the patient and parents regarding the available options, potential risks, and expected outcomes are deemed essential. Identifying reversible causes for the initial flap failure is also emphasized to reduce risks in subsequent procedures. This approach requires a comprehensive assessment of the patient’s medical status aimed at optimizing their candidacy for potential subsequent interventions, with a specific focus directed towards mitigating any underlying pathological factors implicated in the initial flap failure. Given supportive findings for the efficacy of a second free flap for salvage reconstruction, this approach is preferred whenever feasible. Nonetheless, it is crucial to consider individual patient circumstances, including comorbidities and recipient site characteristics. Ultimately, the objective is to achieve optimal outcomes encompassing cosmesis, function, and complication rates, recognizing the need for a tailored approach to maximize success rates in each case [25].

The retrospective design of this study necessitated the use of electronic medical records, which introduces the possibility of substantial data loss due to incomplete documentation from the healthcare providers or variations in medical terminology usage. Additionally, crucial information regarding free-flap dimensions and vasculature diameter was unavailable, potentially impacting the outcomes of free-flap microvascular reconstruction, including the risk of flap failure. The recommendations for further studies underscore the pressing need for standardization in both flap selection and perioperative care for pediatric patients undergoing free-flap microvascular reconstruction. Given the scarcity of studies in the literature in this area, it is imperative that future research prioritizes the development of protocols and guidelines aimed at standardizing the selection of appropriate flaps, surgical techniques, and postoperative care measures. By establishing standardized procedures, the potential for enhancing the overall success rate of these reconstructions and improving outcomes for pediatric patients becomes evident. Additionally, there is a critical need for further exploration into the harmonization of perioperative care, particularly in the realm of anesthetic management for pediatric patients undergoing such procedures. The perioperative period significantly influences complication rates and overall outcomes. Therefore, the implementation of standardized protocols for anesthesia, encompassing preoperative assessment, intraoperative monitoring, and postoperative pain management, is essential for mitigating postoperative complications effectively. Enhanced coordination and consistency in perioperative care have the potential to augment the success rate of reconstructions and contribute to better patient outcomes. Further scientific inquiry of a similar nature is warranted to validate and build upon our findings, ultimately advancing the field and improving patient care practices.

## 5. Conclusions

The aim of our study was to identify key factors influencing the success of maxillofacial microvascular free-flap reconstructions in pediatric and young adult patients. Our findings point towards the importance of the recipient site, particularly the challenges associated with maxillary reconstructions. The lack of significant correlation with age and gender shifts focus to site-specific variables rather than demographic ones. This study, therefore, underscores the need for specialized surgical strategies for maxillary reconstructions in the young population.

## Figures and Tables

**Table 1:** Demographics.

	Frequency	Percent
**Sex**		
Female	76	55.9%
Male	60	44.1%
**Etiology**		
Congenital	39	28.7%
Oncological	82	60.3%
Traumatic	5	3.7%
Iatrogenic	10	7.4%
**Recipient Site**		
Mandible	55	40.4%
Maxilla	56	41.2%
Soft tissue	17	12.5%
Orbit	4	2.9%
Facial nerve	4	2.9%
**Donor Site**		
Iliac crest	44	32.4%
Medial condyle of femur	15	11.0%
Fibula	47	34.6%
Antero-lateral thigh	17	12.5%
Forearm	7	5.1%
Gracilis muscle	4	2.9%
Lower limb nerve	2	1.5%
**Recipient Site Complications**		
Nerve palsy	1	0.7%
Abscess	4	2.9%
Partial flap necrosis	11	8.1%
Total flap necrosis	14	10.3%
None	106	78%
Total	136	100%

**Table 2:** Summary statistics of patient’s age by flap survival or total flap loss.

	Count	Average	Median	Standard Deviation	Minimum	Maximum
Flap survived	122	13.4	14.0	5.0	1.0	25.0
Total Flap Loss	14	14.2	14.5	4.95	4.0	22.0
Total	136	13.5	14.0	4.98	1.0	25.0

**Table 3:** Summary of number of procedures resulting in flap survival or total flap loss with calculated success rate by gender.

Gender	Number of Procedures with Flap Survival	Number of Procedures with Total Flap Loss	Total Number of Procedures	Success Rate
Female	65	11	76	85.5%
Male	57	3	60	95%

**Table 4:** Summary of occurrence of recipient site complications by age group with calculated success rate for each age group.

Age Group	Number of Complications		Total Number of Procedures	Success Rate	Complication Rate
	Other	Total Flap Loss			
Less than 5 y.o.	0	1	8	87.5%	0%
6 to 10 y.o.	2	2	25	92%	8%
11 to 15 y.o.	6	4	39	89.7%	15.4%
16 to 20 y.o.	6	6	56	89.3%	10.7%
Greater than 20 y.o.	2	1	8	87.5%	25%

**Table 5:** Summary of numbers of complications in recipient site with calculated success rate for each recipient site.

Recipient Site	Number of Flap Survival	Number of Total Flap Loss	Total Number of Procedures	Success Rate
Maxilla	45	11	56	80.4%
Mandible	52	3	55	94.6%
Soft tissue	17	0	17	100%
Orbit	4	0	4	100%
Facial nerve	4	0	4	100%

**Table 6:** Summary of number of procedures with and without complications in recipient site.

Recipient Site	Number of Procedures with Complications (Including Total Flap Loss)	Number of Procedures without Complications	Total Number of Procedures
Maxilla	17	39	56
Mandible	12	43	55
Soft tissue	1	16	17
Orbit	0	4	4
Facial nerve	0	4	4

## Data Availability

The data are available upon request from the corresponding author.
